# A Neural Network-Based Weighted Voting Algorithm for Multi-Target Classification in WSN

**DOI:** 10.3390/s24010123

**Published:** 2023-12-26

**Authors:** Heng Zhang, Yang Zhou

**Affiliations:** College of Advanced Interdisciplinary Studies, National University of Defense Technology, Changsha 410073, China; zhnudt@126.com

**Keywords:** WSN, multi-target classification, NN-based classifier, NN-based weighted voting algorithm

## Abstract

One of the most important applications in the wireless sensor networks (WSN) is to classify mobile targets in the monitoring area. In this paper, a neural network(NN)-based weighted voting classification algorithm is proposed on the basis of the NN-based classifier and combined with the idea of voting strategy, which is implemented on the nodes of the WSN monitoring system by means of the “upper training, lower transplantation” approach. The performance of the algorithm is verified by using real-world experimental data, and the results show that the proposed method has a higher accuracy in classifying the target signal features, achieving an average classification accuracy of about 85% when utilizing a deep neural network (DNN) and deep belief network (DBN) as the base classifier. The experiment reveals that the NN-based weighted voting algorithm enhances the target classification accuracy by approximately 5% in comparison to the single NN-based classifier, but the memory and computation time required for the algorithm to run are also increased at the same time. Compared to the FFNN classifier, which exhibited the highest classification accuracy among the four selected methods, the algorithm achieves an improvement of approximately 8.8% in classification accuracy. However, it incurs greater overhead time to run.

## 1. Introduction

WSNs consist of nodes with limited capacity for signal sensing, processing, and wireless communications [1]. Target classification in WSNs plays a critical role in the maintenance of intelligent transportation systems [2] and battlefield environment monitoring [3], while the large amount of sensor data acquired by WSNs and the limited processing capability is the main constraints to target classification in WSNs.

In recent years, researchers have redirected their focus from theoretical studies of target classification in WSNs to practical implementations. In practical applications, particularly in battlefield surveillance settings, limited energy and communication capabilities mean that WSN nodes cannot transmit all collected signal data to the remote monitoring center for target classification. The optimal approach is to perform target classification on the WSN node and transmit only the classification results to the remote monitoring center, enabling energy conservation and the extension of the node’s lifespan.

The process of classifying targets in a WSN involves detecting the target, acquiring data, performing feature extraction on the acquired data, and using a classifier to classify the extracted features of the target signals. In the case of multi-target classification in WSNs, it can be seen as a single-label multi-classification algorithm that has limited computational resources.

Multi-classification methods are categorized into three groups: classical multi-classification methods, single-label multi-classification algorithms consisting of multiple binary classifiers, and neural network-based methods.

The most commonly utilized classical multi-classification algorithms are K-nearest neighbor (KNN) [4], decision tree (DT) [5], Naïve Bayes (NB) classifier [6], and adaboosting algorithm [7]. These algorithms employ their own specific judgment criterion to calculate the input feature data and assign corresponding category labels. The benefit of this algorithm lies in the clear and interpretable judgment criterion, but it suffers from issues such as overfitting and a negative impact on data with high dimensions, ultimately affecting classification accuracy [8].

The second method involves employing multiple binary classifiers to convert a multi-classification problem into several binary classification problems, and the final outcome is determined by the result tree of these classifiers. Typical techniques used for this approach are the OAA-SVM method [9], DAGSVM [10], BTMSVM method [11], and binary-tree SVM method [12]. However, a large number of classifiers requires training, and the presence of unclassifiable data are disadvantages of these algorithms.

The neural network-based method is the third type of approach that has captured the attention of researchers in recent years. This technique postulates that there is a nonlinear correspondence between each target feature and its corresponding category label. After establishing this correspondence through the sample feature data and training the network with determined parameters, the trained network then computes the input feature data to determine the corresponding category label. Wang et al. [13] proposed a powerset fusion network (PFN) method to classify moving targets using acoustic-vibrational signal features. Xu et al. [14] used the parallel recurrent neural network (PRNN) method to classify vibrating targets. Belsare et al. [15] have created a convolution neural network (CNN) trained using preprocessed data for feature extraction and waste image classification in WSN. In addition to this, Kim [16] proposed the Gaussian mixture model (GMM) algorithm for node-level target classification in WSN.

In our prior study [17], we conducted a comparison of three categories of classification methods for classifying vehicle signals in WSNs. The results show that the NN-based algorithm exhibited superior classification accuracy. Nonetheless, their engineering implementation was more intricate, particularly during the network training phase, making them less suitable for node-level application in WSNs.

Therefore, for enhancing classification accuracy of the NN-based classifier, we propose a multi-classifier weighted voting strategy. The experimental outcomes of this strategy, utilizing three classifiers, demonstrate that it substantially improves classification accuracy as compared to a single classifier. Furthermore, we propose an approach for implementing the NN-based classifier guiding by the thoughts of “upper-end training, lower-end transplantation”, to simplify the target classification process of NN-based classifiers into matrix computation. This approach enables the neural network-based multi-classifier weighted voting method to be engineered on WSN nodes with limited resource consumption.

The rest of the paper is organized as follows. Some related work are reviewed in Section 2. Section 3 introduces the proposed multi classifier weighted voting strategy for target classification in details. Based on this, Section 4 describes the engineering implementation process of the NN-based weighted voting algorithm to resource-limited WSN nodes. Section 5 is the discussion of the method’s performance; and the paper is concluded in Section 6.

## 2. Related Work

In this section, we conducted a brief review regarding the effectiveness of different classification algorithms for target classification in previous studies. This section is organized into two parts: performance of different classification algorithms, and NN-based classifier.

### 2.1. Performance of Different Classification Algorithms

In our previous study [17], three groups of classification methods mentioned above were compared for their effectiveness in classifying vehicle targets in WSNs. KNN, DT, NB, AdaBoost, DAGSVM, and NN-based methods were utilized for classifying the vehicle targets, respectively. The classification results indicate that all these methods achieved the purpose of target classification in WSN with differences in performance. The KNN algorithm is the simplest algorithm in terms of implementation, but the classification accuracy is unsatisfactory compared with other algorithms. The NN-based classifier has a higher classification accuracy, but it has a higher memory and computational overhead. Simplification of the classification process of the NN-based classifier is considered to achieve better target classification accuracy while making the algorithm run on WSN nodes with limited resources.

### 2.2. NN-Based Classifier

When applying neural network for target classification, its network structure is considered as a mapping approximation system from input to output. The feedback adjustment characterization of the neural network is used to realize the mapping relationship from input (feature data of the target signal) to output (label of the corresponding target), and then the classifier is constructed.

**Figure 1:**
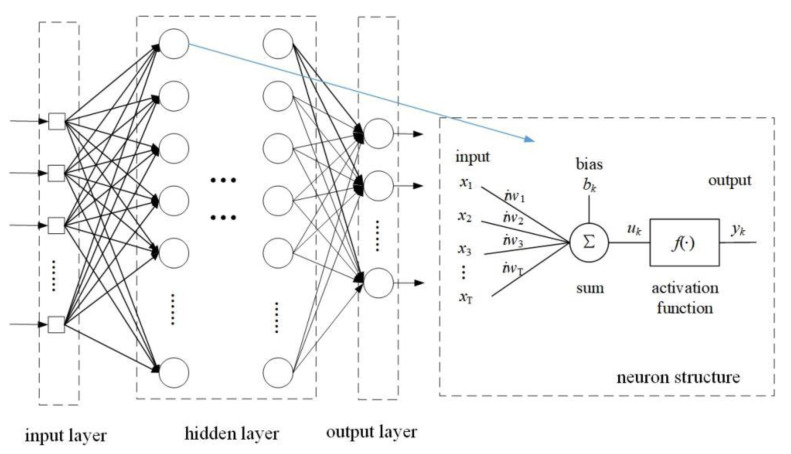
Structure of neural network.

Basheer et al. [18] detailed the structure and application of neural networks. Figure 1 is the typical structure model of the presented neural network and neurons. This model is a nonlinear threshold model that features numerous inputs and a solitary output. Certain symbols are used to represent different elements of the neuron. Specifically, the input signals are denoted by *x_i_*, and the *i*th weight of the neuron is denoted by *iw_i_*. The adder is utilized to calculate the weighted sum of the input signals. Additionally, the bias term of the neuron is represented by *b_i_*, which makes it possible to effectively adjust the range of the neuron’s inputs. Finally, the output of the *i*th neuron is denoted by *y*_i_. An activation function, f(∙), is employed to establish the non-linear relationship between the neuron’s input and output.

Given the input *x_i_*, weights *iw_i_*, bias term *b_i_*, and activation function f(∙) of the neuron described above, neuron *k* can be expressed by the following Equations (1) and (2):

(1) $$u(k)=\sum_{j=1}^{T}x_{i}•iw_{i}+b_{k}$$

(2) $$y(k)=f(u_{k})=f(\sum_{i=1}^{T}x_{i}•iw_{i}+b_{k})$$

where *u*(*k*) represents the weighted sum of neuron inputs and *y*(*k*) denotes output for the *k*th neuron. These equations provide a concise description of a neural model that uses multiple layers of connected neurons to form a neural network for nonlinear map fitting. Theoretically, as long as there is a clear mapping relationship between the input sample feature data and the corresponding category label, the mapping relationship can be fitted by adjusting the parameters of the neurons in the network through network training.

## 3. Multiple Classifiers Weighted Voting Strategy

Using multiple classifiers to vote can effectively improve the accuracy of classification, and the NN-based weighted voting method is described in detail below.

### 3.1. Strategy of Voting

**Figure 2:**
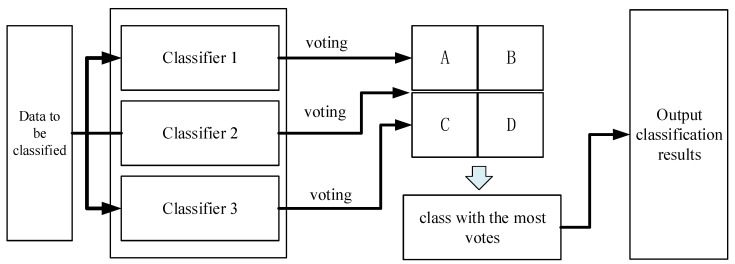
Principles of multi-classifier voting classification methods.

Voting, or Majority Voting [19], is a commonly employed method for combining classifiers. This method is based on the following principle: a set of trained base classifiers (Base Classifier) is used to make classification decisions on the feature data to be classified. Each base classifier casts a vote for the decision result that corresponds to the target class, and the class with the most votes is selected as the classification result for the entire set of classifiers. In this paper, three classifiers are utilized to determine the classification results through a voting process. Figure 2 illustrates the classification process.

Assume that there are *L* base classifiers *C_l_* (l = 1, 2, …, *L*) in the combined classifier, and the output of the each base classifier *C_l_* is a class probability estimate *P*(*f*(**w**) *= k|C_l_*), which represents the magnitude of the probability that the feature data to be classified, **w**, is predicted as class *k* (*k* = 1, 2, …) by the respective classifier *C_l_*. The probability estimates of all base classifiers are combined to calculate the following equation’s value:

(3) $$P(f(w)=k)=\frac{1}{L}\sum_{l=1}^{L}P(f(x)=k|C_{l})$$

The combined classifier determines the probability that the feature vector, **w**, belongs to class *k*, and assigns w to the class with the highest probability value. If all classifiers within the combined classifier classify the feature vector **w** to be classified as an independent event, the classification accuracy of the base classifiers after voting is raised to the P(**w**), where each base classifier *C_l_* in the combined classifier has a classification accuracy of *Pc*.

(4) $$P(w)=1-\sum_{l=0}^{floor(L/2)}C_{L}^{l}{\left(P_{c}\right)}^{l}{\left(1-P_{c}\right)}^{L-l}$$

The function floor(.) rounds downward, while floor(*L*/2) represents the greatest integer not exceeding *L*/2. Take *L* = 3 as an example to illustrate the classification accuracy of the combination classifier, the classification accuracy of the combination classifier is:

(5) $$\begin{array}{ll} P(w) & =1-C_{3}^{0}{\left(P_{c}\right)}^{0}{\left(1-P_{c}\right)}^{3}-C_{3}^{1}{\left(P_{c}\right)}^{1}{\left(1-P_{c}\right)}^{2}\\ & =1-{\left(1-P_{c}\right)}^{3}-3P_{c}•{\left(1-P_{c}\right)}^{2}\\ & =1-{\left(1-P_{c}\right)}^{2}•(2P_{c}+1) \end{array}$$

**Figure 3:**
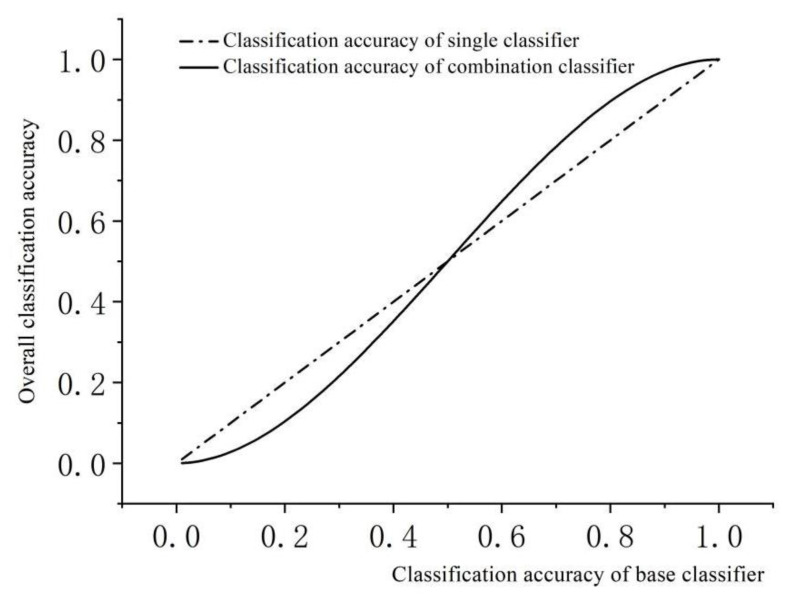
Trend of Combined Classifier Classification Accuracy.

The classification accuracy *P*(**w**) is calculated with *Pc* as the variable, and Figure 3 displays the trends of classification accuracy obtained using a base classifier with 1 and 3 classifiers, respectively.

When base classifiers accurately classify feature vectors over 50%, the accuracy of a combined classifier that utilizes three base classifiers to classify feature vectors through voting is significantly greater than that of a single classifier. The increased voting that takes place after classification by multiple classifiers allows for effective comparison of misclassified feature vectors of individual classifiers and subsequent correction of errors through the application of the “majority rule” principle.

The classification results using the voting algorithm in the best-case scenario without assigning weights to each classifier vote are displayed in Table 1.

When processing the voting results, the category with the most votes is identified as A, and the corresponding target category for the feature data was then also classified as A. However, when the classifier is not set with the voting weights, voting classification using the voting method may result in target categories receiving the same number of votes, leading to an inability to classify them. Table 2 displays an example of such voting results.

As shown in Table 2, the same number of votes were tallied for all three categories: A, B, and D. Consequently, it is unfeasible to determine the category corresponds to the given feature data with the highest votes. To address this predicament, we proposing voting weight values after achieved the classification results from each classifier. If the classification result of a classifier is considered to be more credible, it is given a larger voting weight, whereas a less credible result will be given a smaller weight. Even when multiple classifiers have voting results, as displayed in Table 2, the final vote counts for each class will differ once weights are implemented. The class of the data to be classified can still be successfully determined.

### 3.2. Calculation of Classifier Weights

The primary challenge regarding the incorporation of voting weights is to establish the level of confidence in the classifier’s classification results. The accuracy of classification depends on the performance of the NN-based classifier that has been trained and the features of the data to be classified. The features of the data cannot be controlled, and the classifier’s performance is significantly influenced by the training sample dataset that was used. The principle of classifying neural networks assumes a functional relationship between target feature data and corresponding categories. Sample dataset is used to train the network so that it fits this relationship, processing the feature data for classification.

The mapping accuracy between the feature data fitted by the network and their corresponding classes is higher when there is a large distance between the target feature data of different classes and no data mixing occurs in the sample dataset. The Lance–Williams method [20] is suitable for calculating the distance between sample data for training the classifier. The details of the calculations process is explained in the literature [17].

## 4. Engineering Implementation of the Algorithm

Neural networks require significant computation during training, but the microcontroller processor chips utilized in WSN intelligent monitoring system nodes lack the capability to handle this task. To address this issue, the NN-based voting classification method necessitates the implementation by adopting the “top-end training and bottom-end transplantation” approach. This approach entails training the network on a PC and transplanting its network parameters to the WSN node.

**Figure 4:**
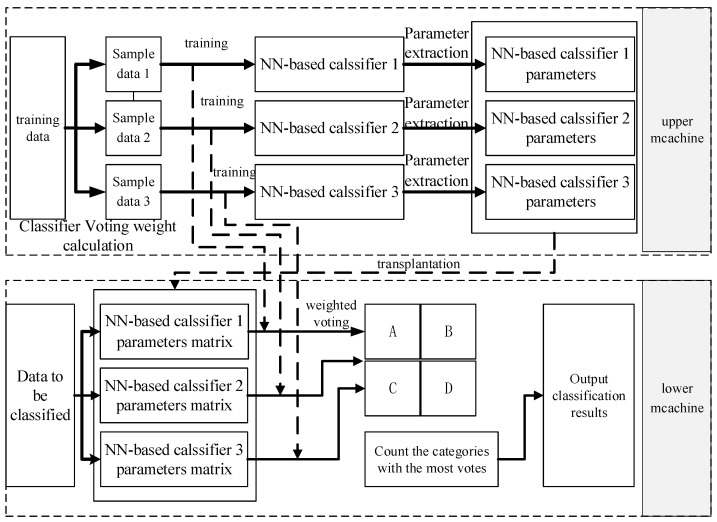
Flow of the Multiple NN-based Classifier Voting Classification Algorithm.

The algorithm’s process is illustrated in Figure 4. Initially, the PC carries out “top-end training” processing, in which using segmented feature data to train NN-based classifiers. Additionally, the voting weights of the classifiers to be identified along with the sample data of the segment are calculated while obtaining the network parameters of several NN-based classifiers at the end of the training. After completing the training, the parameters of NN-based classifiers are acquired, which includes the connection weights and the bias between the neurons. Following that, “down-end transplantation” is performed on the WSN node. The network parameters of NN-based classifiers are exported and stored in the memory chip of the processor unit of the WSN intelligent monitoring system. Lastly, the feature data that requires classification is processed by using the parameters of neural networks. The results of multiple classifiers are utilized to conduct weighted voting classification. Classification results of multiple classifiers obtained by weighted voting process. Then, respective vote scores of categories A, B, C, and D are calculated, and the one with the highest vote score is taken as the final classification result.

The following sections provide explanations for the training of the neural network, transplantation of the NN-based classifier, training data preprocessing, and the flow of the proposed strategy.

### 4.1. Training of Neural Networks

Neural networks are computationally demanding and time-consuming during training, particularly when utilizing lower-performance-embedded processor units. To address this challenge, a common strategy is to train the network on the PC and then transplant it to the embedded processor unit for use.

**Figure 5:**
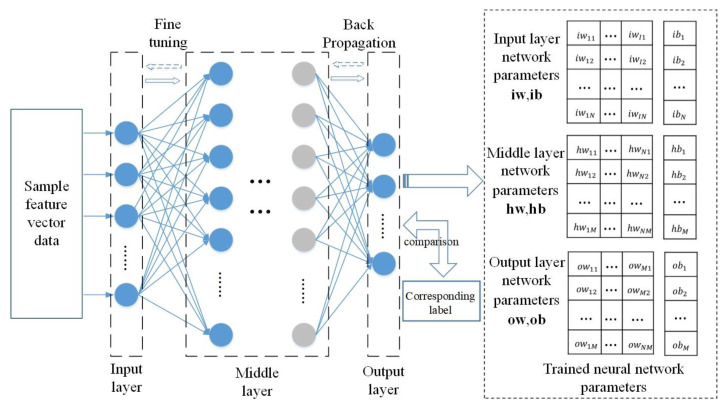
Flow of “Upper Training” in NN-based classifier.

The top-training phase of the NN-based classifier is illustrated in Figure 5. The network is initially trained utilizing training data, and obtained the parameters of the network.

When training a NN-based classifier, predetermined values should be set for the number of layers and number of neurons in each layer. The number of neurons in input and output layers depends on the feature data and the number of target categories. The feature vectors utilized in this study are 16-dimensional, thus requiring 16 neurons in the input layer. The output layer consists of four neurons corresponding to the four target categories (A, B, C, and D). For the four target categories, A, B, C, and D are assigned the labels of (1 0 0 0), (0 1 0 0), (0 0 1 0), and (0 0 0 1), respectively. This allows for the output of 1 to correspond to a neuron in the output layer for each category. The network requires a pre-set number of intermediate layers and neurons in each layer. For this paper, we have set two intermediate layers with 100 and 60 neurons per layer, respectively.

The trained NN-based classifier can be represented by a set of parameters, including the input layer network weight matrix **iw**, input layer neuron bias vector **ib**, intermediate layer network weight matrix **hw**, intermediate layer neuron bias vector **hb**, output layer network weight matrix **ow**, and the output layer bias vector **ob**.

### 4.2. Transplanting of NN-Based Classifier

After training the network, a set of NN-based classifier parameters are settled. These network parameters are exported and deposited into the processor unit on the node of the WSN system to classify the data.

**Figure 6:**
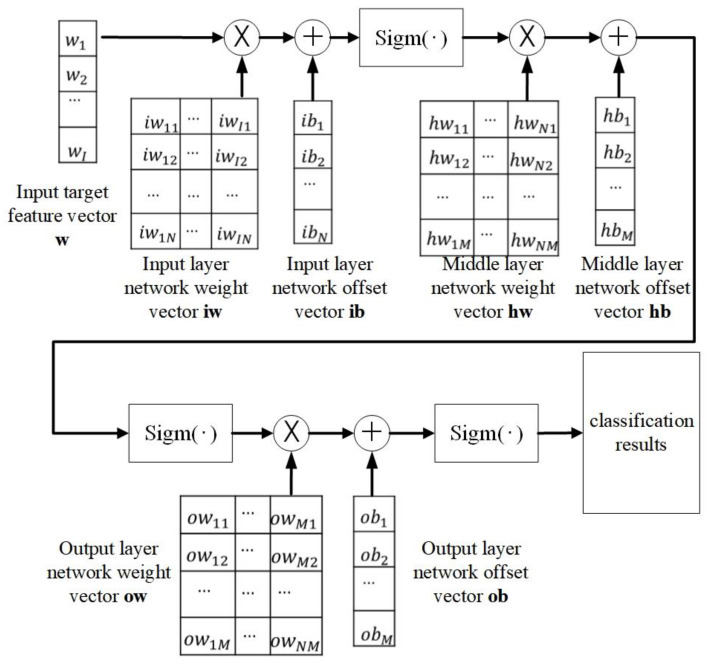
Calculation process using DBN classifier network parameters for classification.

The computational flow for classifying the target data using the settled parameters is illustrated in Figure 6. The feature vector utilized for classification is represented by the symbol **w**. The equation for determining the classification result, denoted as **r**, is displayed in Equation (6).

(6) r=sigm{sigm[sigm(w•iw+ib)•hw+hb]•ow+ob}

where sigm(.) is the selected sigmoid activation function for training, the formula displayed below:

(7) $$sigm(x)=\frac{1}{1-e^{-x}}$$

### 4.3. Training Data Preprocessing

When training the neural network, some training data with large deviations from the mean value may cause misclassification, leading to poorer algorithm performance. Moreover, using the same training dataset to train all three classifiers does not fully optimize the performance of the comparison segment of the algorithm. Thus, the training data is processed using the segmented averaging means to ensure that the dataset for training has a certain degree of variation.

Assuming the feature data for training is a matrix F with N vectors as depicted below:

(8) $$F=\left[\begin{array}{l} f_{1}\\ f_{2}\\ \vdots \\ f_{N} \end{array}\right]=\left[\begin{array}{cccc} w_{11} & w_{12} & \cdots & w_{1m}\\ w_{21} & w_{22} & \cdots & w_{2m}\\ \vdots & \vdots & \vdots & \vdots \\ w_{N1} & w_{N2} & \cdots & w_{Nm} \end{array}\right]$$

The feature matrix is initially partitioned into three sections, namely **FP1**, **FP2**, and **FP3**, as illustrated in the equation below:

(9) $$\begin{array}{l} FP_{1}={\left[f_{1},f_{2},\cdots f_{M}\right]}^{T}\\ FP_{2}={\left[f_{M+1},f_{M+2},\cdots ,f_{2M}\right]}^{T}\\ FP_{3}={\left[f_{2M+1},f_{2M+2},\cdots ,f_{N}\right]}^{T} \end{array}$$

To minimize the impact of outlying values in the training data on the classifier’s performance, we perform an averaging treatment on the three data segments. The first segment remains unchanged, the first and second segments’ data are averaged to create the new second segment data, and the first, second, and third segments’ data are averaged to create the new third segment data. The resulting averaged segment data, **FT1**, **FT2** and **FT3**, are listed below.

(10) $$\begin{array}{l} FT_{1}=FP_{1}\\ FT_{2}=(FP_{1}+FP_{2})/2\\ FT_{3}=(FP_{1}+FP_{2}+FP_{3})/3 \end{array}$$

The main purpose of segmenting the feature matrix of the training data is to create a varied training dataset for training three NN-based classifiers. Additionally, averaging the segmented data aims to eliminate outliers in the training data.

### 4.4. The Flow of the NN-Based Weighted Voting Strategy

The algorithm flow of the NN-based weighted voting strategy is explained in Algorithm 1. It should be noted that the number of parameters in Equation (6) varies with different network structures. As a result, the formula for calculating the classification results based on the network parameters needs to be modified accordingly.
**Algorithm 1**: NN-based Weighted Voting Algorithm  Input: Extracted feature data (including sample feature data F, and the corresponding labels L; and feature data to be categorized CF, category labels is denote as Li (i = 0, …, N), where N is the number of categories).  Output: Corresponding target category label (denote as L)  (1) Preprocessing the training dataset using the segmented averaging means introduced in Section 4.3. The feature data and labels for the processed samples (**FT**1, **FT**2, **FT**3) and (**CF**1, **CF**2, **CF**3) have been acquired.  (2) Three NN-based classifiers are trained utilizing the acquired sample data ((**FT**1, **FT**2, **FT**3), (**CF**1, **CF**2, **CF**3)):  utilizing (**FT**1, **CF**1) for training classifier 1;  utilizing (**FT**2, **CF**2) for training classifier 2;  utilizing (**FT**3, **CF**3) for training classifier 3;  (3) Based on the parameters of the trained NN-based classifier, extract the parameter matrix (**iw**,**ib**,**hw**,**hb**,**ow**,**ob**) for subsequent classification.  (4) The Lance-Williams method, as described in Section 3.2, is utilized to calculate the weights of the three classifiers, identified as WC1, WC2, and WC3. These weights are computed using the sample feature data (**FT**1, **FT**2, and **FT**3) and the target feature data **CF** that is to be classified.  (5) According to the computational formula presented in Section 4.2, the matrices detailing the parameters of the three classifiers are employed in order to compute the feature data to be classified. Subsequently, the classification results of the three classifiers, denoted as r(c1), r(c2), and r(c3), are obtained;  (6) Statistically weighted classification result r is derived from the classifier weights computed in (4):        r=WC1×r(C1)+WC2×r(C2)+WC3×r(C3)  Find the item with the highest weight of the label L, and its corresponding category is the classification result of the input feature data.

## 5. Performance of Multi-DBN Weighted Voting Algorithm

In this paper, the proposed algorithm is validated using the feature data of four targets, which are AAV vehicles, DW vehicles, small domestic vehicles, and pedestrians. Among them, the signal data of AAV vehicles, DW vehicles from are gathered from a real-world experiment, named the third SensIT situational experiment [21]. The details of the experiment were described in the paper mentioned above.

**Figure 7:**
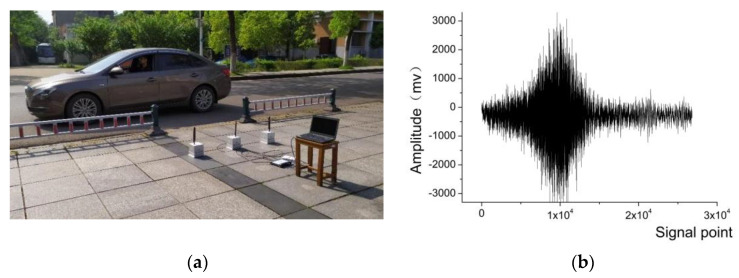
Experimental scenarios for small vehicle and personnel signal acquisition and personnel (**a**) experimental scenario; (**b**) Example of acquired acoustic signal of small vehicle.

The signal of small vehicle and personnel were tested in real-world scenarios using WSN nodes specialized for collecting signals. The sensor nodes were placed on open ground at various distances from the road, with adjustments made gradually. WSN nodes collected sound, vibration, infrared, and magnetic signals from a range of targets including various types of vehicles and individuals. The experiment selects the acoustic and vibration signals obtained from small vehicles and personnel targets. The schematic diagram demonstrating the experiment, presented in Figure 7a,b, shows the acquired acoustic signals of small vehicle.

The data from both AAV and DW vehicles in the SensIT dataset, as well as from personnel and small vehicles in the acquired dataset shown above, are combined to create a multi-target signal dataset for the validation of the proposed classification algorithm. The feature extraction method utilizes the WCER approach, which is described in detail in the paper [22].

The features data are 16-dimensional vectors. From the sensIT dataset, we perform feature extraction on the signals of AAV and DW vehicles, and acquired 9930 feature vectors from the acoustic signals of the AAV vehicle and 10,498 feature vectors from the acoustic signals of the DW vehicle. In total, 726 feature vectors were extracted from the acoustic signal generated by a person, and 879 feature vectors were extracted from the acoustic signal of the small vehicle.

In this study, 3403 AAV feature vectors (34.3%) were utilized to train the classifier, while the remaining 6527 feature vectors (65.7%) were used for testing. Overall, 3205 DW feature vectors (30.5%) were utilized for training, and the remaining 7293 feature vectors (69.5%) were used for testing. Additionally, 230 personal feature vectors (31.7%) were training data, and the remaining 496 feature vectors (68.3%) were testing data. For small vehicle feature vectors, 201 of them (22.4%) were utilized for training, and the remaining 7293 feature vectors (77.6%) were used for testing.

### 5.1. Performance Analysis of Multi-DBN Weighted Voting Algorithm

The SensIT experiment employed AAV vehicles and DW vehicles, while the introduced experiment in Figure 7 utilized personnel and small vehicles as the target to be classified, using part of the selected acoustic signals from four target types and extracting the feature data using the WCER feature extraction algorithm [22]. The target category label corresponding to the AAV vehicle target feature data was set to be {1 0 0 0}; the target category label corresponding to the DW vehicle sample feature data was set to be {0 1 0 0}; the target category label corresponding to the personnel target feature data was set to be {0 0 1 0}; and the target category label corresponding to the small vehicle target feature data was set to be {0 0 0 1}.

Three deep belief networks (DBN) [23] were used as basic classifiers, each with the same network size of one input layer, two intermediate hidden layers and one output layer. The input layer of the classifier network contains 16 neurons, representing the 16-dimensional feature vectors of the target signal. The first hidden layer of the intermediate layer contains 100 neurons, and the second hidden layer contains 60 neurons. The output layer contains four neurons, corresponding to the four different types of targets.

The training data was first segmented and averaged, which was then divided into three equal-sized segments, for training three DBN classifiers. The voting weights for these classifiers were then calculated using the aforementioned sample feature data segments. The three trained classifiers were fed the feature data for calculation and voting classification. The resulting classification was then compared to the original target categories to determine accuracy. After obtaining accuracy for all datasets, the average classification accuracy for each target category was calculated.

Table 3 shows the details of the three classifiers and the final voting results. The voting weights indicate the relative weight of this classifier in performing category voting; 0.8252 is the voting weight of classifier 1, meaning that if classifier 1 identifies a feature data as target A, then target A is assigned a vote value of 0.8252.

According to Table 3, the following conclusions can be drawn from the results of the multi-DBN voting classification method for classifying AAV vehicles, DW vehicles, personnel, and small vehicles.

(1) The proposed NN-based weighted voting strategy can accurately classify different targets in the WSN system, with a classification accuracy of over 70% for the four target categories when using the DBN as the basic classifier. (2) The NN-based weighted voting classification algorithm enhances the overall classification accuracy when compared to the average results of the three individual classifiers for AAV targets. The voting session significantly improves accuracy rates by about 5% when compared to the single DBN classification. The classification accuracy rate for DW targets increased by approximately 5.86%, while the rate for personnel targets increased by 6%. Additionally, the increase in classification accuracy is approximately 6.5% when the NN-based weighted voting classification algorithm utilizes DBN as the basic classifier. For targets of small vehicles, the increase in classification accuracy is approximately 3.49%. (3) Additionally, the classifiers’ performance varies based on the sample data used to train them. For AAV targets, the classification accuracy of classifiers trained using the third segment of sample data after segmentation is significantly lower compared to the first two classifiers. (4) The classification accuracy of classifiers for both AAV and DW targets is generally low, which can be attributed to their lower accuracy for both target types. Classification accuracy is generally low because the acoustic and vibration Seismic of AAV targets and DW targets are similar, and it is more difficult to distinguish the feature data.

### 5.2. Effect of Network Size on Algorithm Classification Accuracy

For studying how the size of network affect the classification accuracy of NN-based weighted voting strategy, we utilized neural networks of varying size as the basic classifiers to classify the target data.

(M, N) were used to represent the dimensions of the hidden layer in the DBN classifier. Here, M refers to the number of neurons in the first layer, while N indicates the number of neurons in the second layer. We utilized the DBN classifiers with scales (100, 40), (100, 60), (100, 80), (200, 60) and (300, 60) to classify the targets, respectively.

**Figure 8:**
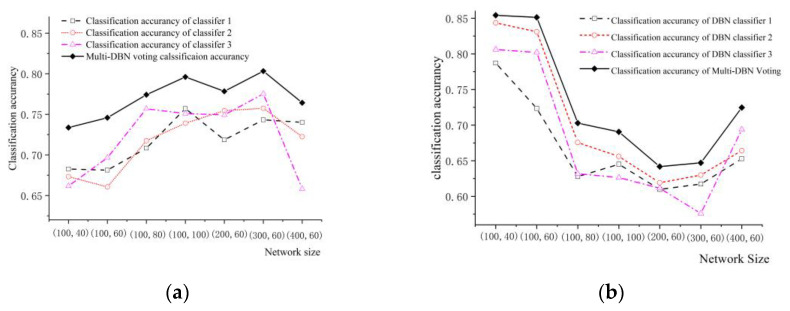
The effect of network size on classification accuracy: (**a**) Classification Accuracy of Algorithms for AAV Vehicles in Different Network Sizes; (**b**) Classification Accuracy of Algorithms for DW Vehicles in Different Network Sizes.

The classification accuracy of three DBN classifiers and NN-based weighted voting algorithm were computed. The classification results for both AAV vehicles and DW vehicles are illustrated in Figure 8.

As illustrated in Figure 8, from the classification accuracies of the single classifier and the voting strategy at various network sizes, we can draw the following conclusions:

(1) The proposed voting classification strategy yields higher accuracy than the highest accuracy achieved by the three single classifiers. Additionally, there is no discernable correlation between network size and classification accuracy of the DBN classifier, larger networks do not necessarily result in higher accuracy.
(2) The classification accuracy of DW vehicles decrease as the network size increases. Additionally, the DBN classifier’s classification accuracy for AAV vehicles is negatively correlated with the network size. When the classifier’s accuracy for AAV vehicles improves, the corresponding accuracy for DW vehicles decrease.

Since there is no direct relationship between the size of the neural network and overall classification accuracy. Therefore, it is important to comprehensively consider the actual application scenario of the method to select the appropriate network size. As the network size increases, the NN-based weighted voting strategy requires more memory and computation source.

### 5.3. Comparison with Other Multi-Objective Classification Methods

In addition to deep learning algorithms, (KNN) [24], the Plain Bayes (NB) method [25], and the Extreme Learning Machine (ELM) [26] algorithms are also well-established classical methods for multi-classification. These centralized methods were applied to classify the four target signals in the WSN monitoring system and compare the results with those of the NN-based weighted voting methods.

The average classification accuracy is noted as OAc, the formula is presented as follows:

(11) $$OAc=\frac{1}{P}\sum_{i=1}^{P}Ac(i)$$

where *Ac*(*i*) is the classification accuracy of the *i*th class, and *P* is the number of target class.

Table 4 shows the overall average classification accuracy of each classification algorithm for four kinds of targets.

where the average classification time is determined by adding the classification time for all data records in the dataset and dividing by the total number of data records. Based on the overall average classification accuracy results of each algorithm for the four targets presented in the table, we can draw the following conclusions:

(1) The NB method yields a lower average classification accuracy of approximately 63.85% when used to classify multiple vehicle signals detected in the WSN system. The main reason for this is that when the sample training dataset is small, the a priori probability model, obtained by analyzing the training data using the Naive Bayes (NB) method, is not accurate.
(2) Additionally, when classifying multiple target signals using the Adaboosting method, the accuracy is lower compared to other methods.
(3) When classifying multiple vehicle signals detected in the WSN monitoring system, the neural network-related methods (such as the multi-DBN voting method and FFNN method) exhibit higher average classification accuracy. Moreover, the neural network method’s advantage in classification accuracy becomes more apparent after the network optimization.
(4) The NN-based weighted voting method achieves the highest classification accuracy, although its classification time is the longest compared to other methods.

When implementing the multi-DBN neural network using the “top-end training, bottom-end transplantation” approach, the main memory consumption occurs during the top-end training phase. The memory consumption of bottom-end transplantation is dependent on network parameters. In this paper, we used a four-layer network for the DBN classifier with a total of 280 neurons, divided into 16, 100, 60, and 4, respectively. The resulting network parameters consist of six matrices, sized 16 × 100, 16 × 1, 100 × 40, 100 × 1, 40 × 4, and 40 × 1, respectively, occupying approximately 11.8 kB of memory space.

### 5.4. Comparison of Classification Performance Using Different Neural Networks

For comparing the classification performance using different neural networks on target classification, we employed the Feedforward neural network (FFNN) [27], deep neural network (DNN) [28], and DBN as the base classifiers of the NN-based weighted voting algorithm. All the neural networks have 16 neurons in the input layer and 4 neurons in the output layer. FFNN includes one hidden layer with 100 neurons, while DNN includes three hidden layers with 100 neurons in each hidden layer. The classification accuracy of using the FFNN, DNN, and DBN are summarized in Table 5.

Based on the classification accuracies tabulated using different types of neural networks as base classifiers, we can draw the following conclusions:

(1) The neural network-based weighted voting algorithm yields varying classification results when employing different neural networks.
(2) Classification accuracy significantly improves when using DNN and DBN classifiers compared to FFNN, with an achievement of about 85%.

## 6. Conclusions

In this paper, a novel NN-based weighted voting strategy on the basis of single NN-based classifier and combined with the idea of voting method for multi-target classification in WSNs is proposed. The introduced approach uses multiple NN-based classifiers cooperating with each other to classify multi targets. According to the training and classification characteristics of neural networks, we propose the means of “top-end training, bottom-end transplantation” for transplanting the NN-based weighted voting classification method to the WSN node. The experiment results reveal that the proposed method has a higher accuracy in classifying the target signal features, achieving an average classification accuracy of about 85% when utilizing DNN and DBN as the base classifier. Moreover, the NN-based weighted voting algorithm enhances the target classification accuracy by approximately 5% in comparison to the single NN-based classifier.

However, there are still several issues that necessitate further investigation.

(1) A more thorough investigation of classification accuracy among algorithms using various neural networks to determine the optimal neural network structure.
(2) The simulations show a significant impact of different training sets on the classification performance of neural networks, and more studies are needed to reveal the underlying patterns.
(3) The size of the neural network, particularly the number of hidden layers, can impact the classifier’s classification accuracy. However, augmenting the number of hidden layers increases computing power demands, and finding a balance requires further research.

## Figures and Tables

**Table 1:** Classification results of the multi-classifier voting method in the ideal case.

Title 2	Classifier 1 Voting	Classifier 2 Voting	Classifier 2 Voting	Voting Results
A	1	1	0	2
B	0	0	0	0
C	0	0	0	0
D	0	0	1	1

**Table 2:** Classification Results of Multi-Classifier Voting Methods in Extreme Cases.

Title 2	Classifier 1 Voting	Classifier 2 Voting	Classifier 2 Voting	Voting Results
A	0	1	0	1
B	1	0	0	1
C	0	0	0	0
D	0	0	1	1

**Table 3:** Multi-DBN classifier classification accuracy of different targets.

**Classifier**	**Classifier 1**	**Classifier 2**	**Classifier 3**	Voting Classification Results
Voting Weighted Value	0.8252	0.7839	0.7613	
Classification accuracy of AAV	68.12%	66.06%	69.65%	73.37%
Classification accuracy of DW	80.21%	83.11%	72.35%	85.42%
Classification accuracy of personnel	87.24%	84.63%	81.18%	90.76%
Classification accuracy of small vehicle	68.12%	66.06%	69.65%	88.96%

**Table 4:** Comparison of classification effects of different algorithms.

Algorithm	KNN	NB	ELM	NN-Based Weight Voting
accurancy	61.71%	63.85%	71.88%	84.63%
time consumption (s)	0.0043	0.0077	0.1783	0.4892

**Table 5:** Comparison of classification effects using different NN-based classifier.

Neural Network Types	DBN	FFNN	DNN
Average classification accuracy	84.63%	75.76%	85.49%

## Data Availability

This work used data publicly available from [21].

## Abbreviations

The following abbreviations are used in this manuscript

WSNs	Wireless sensor networks
NN	Neural network
DBN	Deep belief network
FFNN	Feedforward neural network
DNN	Deep neural network
WCER	Wavelet coefficient energy ratio
AAV	Assault amphibian vehicle
DW	Dragon wagon
KNN	K-nearest neighbor
ELM	Extreme Learning Machine
NB	Naive Bayes
